# Effects of replacing commercial zinc bacitracin with insect meal (*Macrotermes subhylanus*) on caeca bacteria composition, haematology, and growth performance in commercial broiler chicks at the starter phase

**DOI:** 10.1016/j.psj.2026.107218

**Published:** 2026-06-02

**Authors:** Emmanuel Malematja, Thendo Mafuna, Nthabiseng Amenda Sebola, Sekobane Daniel Kolobe, Monnye Mabelebele

**Affiliations:** a University of South Africa, Department of Agriculture and Animal Health, College of Agriculture and Environmental Sciences, Florida, 1710, South Africa; b University of Johannesburg, Department of Biochemistry, Auckland Park, South Africa

**Keywords:** Microbiome composition, Insect meal, Gut microflora, Blood profile, Apparent digestibility

## Abstract

The effects of *Macrotermes subhylanus* meal as a substitute for zinc bacitracin on the composition of caecal bacteria, haematology, and performance parameters were evaluated in broiler chicks during the starter phase. Three isoproteic and isoenergetic diets, NC (negative control; basal diet only), PC (positive control; basal diet with 0.05 g of zinc bacitracin per kg DM) and InsecM (basal diet with 100 g of insect meal per kg DM), were used in this study. A total of 150 one-day-old broiler chicks were randomly assigned to each of the 3 treatments, with 10 chicks per replicate and 5 replicates per treatment in a completely randomised design. Data on bacterial composition, apparent nutrient digestibility, blood profiles, and growth parameters were measured on day 14 and analysed using DADA2 and SAS software for statistical purposes. The results revealed that Firmicutes was the predominant (P < 0.05) phylum, proportionate to Actinobacteriota, Bacteroidota, Cyanobacteria, Proteobacteria, Verrucomicrobiota, and Vertebrata in chicks fed an InsecM diet compared to those on NC and PC diets. Furthermore, the NC diet reduced (P < 0.05) apparent dry matter (83.2%) and crude protein, while crude fibre digestibility (75.62%) was significantly lower in the InsecM diet. The apparent crude protein (CP) digestibility on the InsecM diet was significantly improved (86.24%) when compared to the NC and PC diets. Plasma enzyme alanine aminotransferase was also significantly reduced by an InsecM diet (0.87 U/L). However, uric acid (0.27 mmol/l) was elevated (P < 0.05) in chicks fed an InsecM diet. Body weight gain (483.28 g/bird) and the feed conversion ratio (1.12) were improved in chicks fed the InsecM diet. It is concluded that broiler chicks on a diet containing 100 g of *M. subhylanus* meal per kg DM and those on a diet with 0.05 g zinc bacitracin improved caecal bacterial composition, CP digestibility, and growth performance, without causing negative effects on the birds' metabolic pathways and health status. Nonetheless, further studies are encouraged to confirm these findings.

## Introduction

The detrimental health effects of indiscriminate use of commercial antibiotic growth promoters (AGP) in food-producing animals are the main reason why alternative and/or substitute growth enhancers should be investigated (Abreu et al., 2023). Antibiotic growth promoters are synthetic substances developed to alter the growth and survival of pathogenic microorganisms in host animals and are also used to prevent or treat associated diseases (Rahman et al., 2022). Zinc bacitracin is among the conventional AGPs commonly used in the poultry sector to safeguard the health and enhance the growth of birds (Rahman et al., 2022). Zinc bacitracin is an antibiotic comprising a mixture of high-molecular-weight polypeptides of bacitracin derived from *Bacillus lucheniformis* and *Bacillus subtilis*, combined with zinc (Thema et al., 2019). This combination is primarily effective against a range of Gram-positive bacteria and helps maintain healthy gut microbes required for optimal digestion and nutrient absorption, thereby reducing gastrointestinal infections (Engberg et al., 2000; Abreu et al., 2023). However, as the spectrum of diseases in poultry keeps changing and new antibiotic-resistant microbes continue to emerge, the probability and frequency of disease-causing pathogens also keep increasing (Chia et al., 2021).

Previous studies that investigated the evolution of disease-causing pathogens in birds have observed that most pathogens have mutated and developed resistance to AGPs (Manyi-Loh et al., 2018; Abreu et al., 2023). In addition, concerns about the availability and ineffectiveness of AGPs in poultry diets for controlling and preventing contagious diseases present a major barrier for poultry producers (Manyi-Loh et al., 2018). On the other hand, the World Health Organisation (WHO, 2018) has classified the use of AGPs in food-producing animals as a global concern due to antibiotic residues in meat products, which threaten human health (Pokharel et al., 2019; Thema et al., 2019). Consequently, the European Union and other nations have banned the use of synthetic AGPs in food-producing animals (Castanon, 2007). However, some countries, including South Africa and other African countries, still use AGPs in livestock, primarily because of high infectious disease incidence, and research on potential substitutes for conventional AGPs remains lagging in these regions (Thema et al., 2019). Against this backdrop of growing restrictions on AGPs' usage, there is a need for sustainable alternatives.

Given these concerns, the potency of non-antibiotic antimicrobial agents and growth enhancers, such as chitin-derived prebiotics, warrants further investigation. In recent years, the use of insect larvae as a substitute for conventional feed ingredients for poultry has gained global popularity (Hua et al., 2019; Chia et al., 2021; Mabelebele et al., 2023). Edible insect larvae have been targeted for their chitin and are regarded as a promising source of bioactive compounds with positive health benefits, including antimicrobial, anti-inflammatory and antioxidant activities, immunomodulatory effects, gut microflora modulation, and growth-enhancing properties (Józefiak and Engberg, 2015; Biasato et al., 2018; Gasco et al., 2018). It has been demonstrated that chitin-derived prebiotics from insects can be used across various poultry species to stimulate gut development, microflora, and immune responses (Józefiak and Engberg, 2015; Enoka et al., 2020). However, the available literature reported more on growth performance in broiler chickens (Bovera et al*.,* 2015; Amobi et al., 2020; Benzertiha et al., 2020; Elahi et al., 2020), with little being reported on other parameters, such as gut health and haematology. Furthermore, a research gap exists, as less attention has been paid to the utilisation of other insect species, such as termites, and their subsequent effects on gut health, nutrient digestion and absorption, and growth performance in poultry, as this has not yet been explored. Taking advantage of chitin-derived bioactive compounds, their potential utilisation as non-antibiotic gut microflora modulator, immunomodulatory, growth enhancer and/or antimicrobial agent in broiler chicks has received inadequate attention.

With this in mind, this comparative study was carried out to compare the effects of zinc bacitracin (commercial AGP) and an insect-based diet (defatted alate termites of the genus *M. subhylanus*) meal as a non-antibiotic gut microflora modulator, immunomodulatory, and growth enhancer in commercial Ross 308 broiler chicks during the starter phase, as this is a critical stage where most of the development in broiler chicks occurs (Bovera et al., 2015).

## Materials and methods

### Study site

The feeding trial was conducted on a poultry facility in Zuurbekom, Westonaria, in the Gauteng province, South Africa. The study was conducted in winter, when the temperature in the study area ranged between 7.45- 24.95°C (South African weather services, 2010). The chemical and secondary metabolites analysis of the alate termites *M. subhylanus* meal and blood analysis were conducted at the University of Pretoria and Stellenbosch University, while bacteria composition analysis was done at Inqaba Biotech. The protocols employed in the rearing of broiler chickens were confirmed by the guidelines for the care and use of research animals. They were approved by the University of South Africa, College of Agriculture and Environmental Sciences (UNISA-CAES) Animal Research Ethics Committee (2022/CAES AREC/149).

### Preparation of *M. subhylanus* meal and diet formulation

The alate termites of *M. subhylanus* were collected from their natural habitat, euthanised using the thermal immersion method, and their wings were removed before air-drying with no direct sunlight. The dried alate termites were partially defatted using an oil presser machine and thereafter ground into powder for nutrient profiling and diet formulation. A preliminary chemical analysis of the insect meal was quantified, in accordance with the guidelines of the Association of Official Analytical Chemists (Horwitz and Latimer, 2005). The nutritional composition of the *M. subhylanus* meal is shown in Table S1. On proximate analysis, the *M. subhylanus* meal contains 95.80% dry matter, crude protein of 53.62%, crude fibre (6.83%), ether extract (22.43%), 9.68% of ash, and neutral detergent fibre (NDF) of 27.23, respectively. Three isoproteic and isoenergetic diets, in crumble form (starter phase), were formulated by either adding 0.05 g of zinc bacitracin per kg DM or 100 g of *M. subhylanus* meal per kg DM to a complete commercial broiler starter diet and/or neither of the two feedstuffs (Table 1). The formulated diets met the nutritional requirements of Ross 308 broiler chickens, as recommended by Ross (2019). The experimental diets were formulated as follows: (1) Negative control (NC)= commercial broiler diet without zinc bacitracin and insect meal (*M. subhylanus*); (2) Positive control (PC)= commercial broiler diet with 0.05 g of zinc bacitracin per kg DM; (3) Insect-based diet (InsecM)= a commercial broiler diet with 100 g of *M. subhylanus* meal per kg DM.

**Table 1:** Experimental diet composition in percentages.

Composition (%)	Experimental diets		
	NC	PC	InsecM
Maize yellow fine	52.22	52.10	48.89
Soyabean meal 46.5%	20.85	20.68	17
Sunflower oilcake 34%	10.75	10.77	2
Soya full-fat meal	8.55	8.77	0
Crude soya oil	3	3	0.13
Feed line fine	1.17	1.17	1.36
*M. subhylanus*	0	0	10
Wheat bran	0	0	17
Pellibond (Pellet binder)	1	1	1
L-Lysine HCL	0.47	0.47	0.60
DL-Methionine	0.17	0.17	0.20
L-Threonine	0.20	0.20	0.30
L-Tryptohan	0	0	0
L-Vline	0.09	0.09	0.03
MDCP	0.88	0.88	0.94
Salt fine	0.20	0.20	0.18
Sodium Bicarbonate	0.14	0.14	0.12
Zinc bacitracin 15% (AGP)	0	0.05	0
AxtraPhy broiler (100FTU) Phytase	0.01	0.01	0.01
†Broiler starter premix	0.30	0.30	0.30
**Total**	100	100	100

Treatments: Negative control (NC)= commercial broiler diet, without antibiotics growth promoter (AGP) and insect meal (*M. subhylanus*); (2) Positive control (PC)= commercial broiler diet, with zinc bacitracin (AGP) at 0.05 g; and (3) Insect-based diet (InsecM)= a commercial broiler diet, with 100 g of insect meal per kg DM.

† Broiler starter premix: The ingredients contained in the vitamin-mineral premix were as follows (per kg of diet): vitamin A 12000 IU, vitamin D3 3500 IU, vitamin E 30.0 mg, vitamin K3 2.0 mg, thiamine 2 mg, riboflavin 6 mg, pyridoxine 5 mg, vitamin B12 0.02 mg, niacin 50 mg, pantothenate 12 mg, biotin 0.01 mg, folic acid 2 mg, Fe 60 mg, Zn 60 mg, Mn 80 mg, Cu 8 mg, Se 0.1 mg, Mo 1 mg, Co 0.3 mg, I 1 mg.

### Animal management and experimental design

The study involved a total of 150-day-old unsexed Ross broiler chicks (with an average initial live weight of 50 ± 5 g/ bird), purchased from National Chicks Brits, in the Northwest province, South Africa. The house was cleaned and disinfected thoroughly. Upon arrival, the chicks were weighed, blocked by weight, and randomly assigned to 3 dietary groups, each with 5 replicates and 10 chicks per replicate/ pen. The experiment was conducted in a completely randomised design, with each pen as the experimental unit. The birds were reared on floor pens (1.2 m W × 1 m L × 0.8 m H), bedded with wood shavings, in an environmentally controlled house for 14 days. The house temperature was kept at 32°C during the first few weeks and adjusted according to their ideal temperature as the birds grew, and they were subjected to similar husbandry management practices. The chicks were offered feed and water ad libitum*,* and the photoperiod was 23L:1D. All pens were checked regularly for sickness and mortality.

### Data collection

#### Determination of bioactive compounds in the *M. subhylanus* meal

Bioactive compound contents of the *M. subhylanus* meal were determined according to the method described previously by Nino et al. (2021). Approximately 35 µL of extract per sample was mixed with 150 µL of 1 N Folin reagent and allowed to react for 5 min in the dark condition (at room temperature) in a 96-well plate. Furthermore, Na_2_CO_3_ solution (7.5%[W/v]) was added at a rate of 115 µL, and the microplate was incubated at 40°C for 30 min. After the incubation period, the microplate was allowed to cool, and the absorbance at 765 nm was measured through a microplate photometer (Multiskan™ FC Microplate Photometer, Waltham, MA, USA). The results were expressed as gallic acid equivalents per 100 g of extract sample. Furthermore, extracts were resolubilised with 0.5 mL solution of formic acid (0.1%), dd-water (49.9%) and methanol (50%). Thereafter, it was centrifugated, filtered with 0.45 µL PTFE filter, and the supernatant was transferred into a glass vial and readied for liquid chromatography-electrospray ionisation-quadrupole time-of-flight mass spectrometry (LC-MS) analysis, using a Waters Synapt G2 Quadrupole Time of Flight (QTOF) mass spectrometer (MS) connected to a Waters Acquity UPLC system (Waters, Milford, MA, USA), as described previously by Manyelo et al. (2020).

#### Sample collection for the bacteria composition analysis

At the end of the starter phase (d 14), 2 chicks per replicate were randomly selected and humanely slaughtered, and the gut organs were immediately removed. Caeca samples were removed from the digestive system and stored in tubes, filled with 70% ethanol, kept in ice, and transported to the Inqaba laboratory, where they were stored at −20 ⁰C, for DNA extraction and sequencing.

#### DNA extraction and 16 s rDNA polymerase chain reaction amplification sequencing

Briefly, total bacterial community genomic DNA samples were polymerase chain reaction (PCR) amplified using barcoded universal primer pair 27F and 1492R (12 × 27F barcoded primers and 8 × 1492R barcoded primers), targeting the V1 -V9 region of the bacterial 16S rRNA gene. The resulting barcoded amplicons were individually quantified, pooled equimolar, and ampure PB bead-based purification step was performed on the pool. The pooled sample was then used to prepare SMRTbell library following manufacture protocol. The SMRTbell library was qualified using Fragment Analyzer (Agilent) and quantified using Qubit HS dsDNA Assay (Thermo Fisher). The annealing of the sequencing primer and polymerase binding steps was performed following PacBio SMRT link v13.1 software protocol (browser-based) to prepare the library for sequencing on the PacBio Revio system using SMRT cell 25 M. The DNA sequence generated in the present study is accessible in the NCBI Sequence Read Archive under accession ref: PRJNA1237245.

#### Nutrient digestibility

Apparent nutrient digestibility (AND) trials of an insect-based diet compared to a zinc bacitracin diet were conducted at 14 days. Two birds per replicate were randomly selected and placed in individual metabolic cages specifically designed for faecal collection. The cages were equipped with wire mesh floors to facilitate the separation of excreta from feed particles and to prevent contamination. Prior to the collection period, the birds were given three days of adaptation to allow them to adjust to the metabolic cages and their diets, respectively. Apparent digestibility on dry matter (DM), ash, crude protein (CP), crude fibre (CF), ether extracts (EE), and metabolisable energy (ME) were determined, following the total excreta collection. Fresh excreta were collected during the last four days, in the morning, and subsequently stored at – 20°C until freeze-dried (Khalil et al., 2024). Dried samples were ground into a homogenous mixture (particle size of 0.5 mm) and stored at room temperature. Four representative samples were taken for the laboratory analysis.

#### Chemical analyses and calculations

The *M. subhylanus* meal, feeds and excreta samples were analysed for DM, CF, EE, gross energy (GE) and nitrogen (N), according to the AOAC standard procedure (Horwitz and Latimer, 2005). The DM content was determined by method no 930.15. The nitrogen content of the *M. subhylanus* meal, feeds, and excreta was analysed by combustion, using a carbon nanoashere-200 carbon, N and sulphur auto-analyser (MAX N exceed, Elementar, Donaustraze, Hanau, Germany), following the guidelines described by Horwitz and Latimer (2005), method no 968.06. The AP CP was then calculated by multiplying the percentage of N by a correction factor of 6.25, as previously described by Khalil et al. (2024). The EE content was determined by method no 2003.06 of the Horwitz and Latimer (2005), using a Soxhlet extractor (Soxtec System HT 1043 Extraction Unit, Höganäs, Sweden). The ash content was assayed by method no 942.05 of the Horwitz and Latimer (2005), using a muffle furnace, at 550°C for 24 h. Ashed samples were digested with hydrochloric acid for mineral analysis (calcium and phosphorus in the feeds) using method 968.08D, which involved inductively coupled plasma-optical emission spectroscopy with a Thermo Jarrell Ash IRIS instrument (Franklin, MA, USA). Furthermore, fibre analyser (ANKOM Technology, New York, USA) was used to determine the CF of the feed and faeces. The GE was determined using a bomb calorimeter (Gallenkamp Autobomb, Weiss Gallenkamp Ltd, Loughborough, UK) according to the procedure described by Khalil et al. (2024). The obtained GE values were used to calculate the apparent metabolizable energy (ME).

#### Calculations

Apparent nutrient digestibility for dry matter, crude protein, and minerals was calculated using this formula:

$$Apparent\;nutrient\;digestibility=\frac{Nutrient\;intake-Excreta\;nutrient}{Nutrient\;intake}\times \;100$$

$$Apparent\;ME\;\left(MJ/kg\right)=\frac{GE\;intake-GE\;excreted}{Feed\;intake}$$

#### Broiler’s production parameters

The initial weight (IW) of each chick was determined at the start of the feeding trial and thereafter taken weekly as live weight (LW) using an electronic weighing balance (AFP 110 L). Additionally, the obtained IW and LW values were used to calculate the weight gain (WG) of the chick (g) by subtracting IW from LW and the obtained difference was referred to as WG. Feeds were provided in trays and offered to chicks daily, and the refusals were collected and weighed to compute feed intake (FI) by subtracting the sum of feed refusals from the total amount of the feed offered. Furthermore, the FI and WG were used to determine the feed conversion ratio (FCR) of each chick as a fraction of FI and WG of a live chick, as shown below.

$$FCR=\frac{FI\;\left(g\right)}{WG\;\left(g\right)}$$

#### Blood indices and serum parameters

Blood was collected on d 14, and prior to blood collection, 2 chicks per replicate were randomly selected, and deprived feeds for 12 hours to avoid sample contamination; however, water was provided. Hematological samples (complete blood count) were collected from the wing vein of the chicks into tubes containing an anticoagulant. Serum samples were also collected into tubes without anticoagulant for biochemical analysis.

### Statistical analysis

#### Total bacteria analysis

A total of 9 Full 16S rRNA gene samples were used for analysis. The DADA2 pipeline (v1.16.0) implemented in R (v4.1.2) was used to generate an amplicon sequence variant (Callahan et al., 2016). The filter and Trim function of the DADA2 pipeline was used to remove primers. The default settings were used for sequence filtering, trimming, error rate learning, dereplication, chimera removal, and amplicon sequence variant (ASV) inference. The SILVA (v138.1) database was utilised for taxonomic assignment (Gurevich et al., 2013) using the DADA2-formatted training files for taxonomy and assignment up to the genus level (Callahan et al., 2016). The clustered ASV were then rarefied to even sampling depths, and both alpha and beta diversities were calculated. Alpha diversity was calculated using the Shannon index, Simpson index, and observed OTUs index, using the phyloseq package (R version 3.5.0, 2018-04-23). To compute the microbial beta diversity, dissimilarity analyses were performed, and sample Bray-Curtis distances were visualised on PCoA plots. Multivariate beta diversity analysis was verified using non-parametric PERMANOVA and the Adonis function in R with 999 permutations (*P* *<* 0.05). Krona plots were generated from phyloseq objects using the plot_krona function from the psadd R package. Taxonomic abundance data were grouped according to sample metadata variables and visualised as interactive hierarchical charts using KronaTools.

#### Nutrient digestibility, growth, and blood parameters

Collected data on nutrient digestibility, growth performance, and blood parameters were analysed using a one-way analysis of variance (PROC GLM; SAS, 2019) with dietary treatment as the only factor.

$$y_{\text{ij}}=\;\mu +\;T_{i}+\;e_{\text{ij}}$$

where $y_{\text{ij}}=\;$response variables; μ=general mean; $T_{i}=\;$fixed effect of the $i^{\text{th}}$ treatment (i =NC, PC, and InsecM); and $e_{\text{ij}}=$ random residual error.

For all data, significance was set at *P* *<* 0.05, where there were significant differences (*P* *<* 0.05) between the treatments. Tukey’s honestly significant difference test (HSD) was used for mean separation.

## Results

The analysed proximate and mineral composition of the experimental diets is presented in Tables S1 and S2.

### Qualitative results of antimicrobial compounds and growth enhancers bioactive compounds identified in the *M. subhylanus* meal

The results of qualitative bioactive compounds identified in *M. subhylanus* meal are shown in Table 2. A total of thirteen (13) known bioactive compounds, in different classes, were identified in the *M. subhylanus* meal. Among identified compounds, three (3) compounds, namely Hyacinthacine C1; (+)-Hyacinthacine C1, N—Hydroxyannomontine, Floripavidine belonged to the class alkaloids, while four (4) compounds, Phellodendric acid A; (+)-Phellodendric acid, Umbilicaxanthoside A, Dracunculifoside K; (-)-Dracunculifoside K, and Jioglutolide were identified as phenolics. Furthermore, compounds 2-[(5‑hydroxy-2,2-dimethyl-4-oxo-3,4-dihydro-2H-1-benzopyran-7-yl) oxy]-N-[2-(2-methyl-1H-imidazol-1-yl) ethyl] acetamide, Rosmarinic acid, Annularin G;(+)-Annularin G, Prenyl glucoside were identified as flavonoids. Lastly, the compounds kynurenic acid and 2-oxo-1,2-dihydroquinoline-4-carboxylic acid were identified as quinolines.

**Table 2:** Bioactive compounds identified in the *M. subhylanus* meal.

Metabolites group	Molecular formula	Proposed compound
**Alkaloids**		
1	C_9_H_17_NO_5_	Hyacinthacine C1;(+)-Hyacinthacine C1
2	C_15_H_11_N_5_O	N-Hydroxyannomontine
3	C_24_H_29_NO_6_	Floripavidine
**Flavonoids**		
1	C_19_H_23_N_3_O_5_	2-[(5‑hydroxy-2,2-dimethyl-4-oxo-3,4-dihydro-2H-1-benzopyran-7-yl) oxy]-N-[2-(2-methyl-1H-imidazol-1-yl) ethyl] acetamide
2	C_18_H_16_O_8_	Rosmarinic acid
3	C_9_H_14_O_4_	Annularin G;(+)-Annularin G
4	C_11_H_20_O_6_	Prenyl glucoside
**Phenolics**		
1	C_9_H_16_O_5_	Phellodendric acid A;(+)-Phellodendric acid A
2	C_25_H_28_O_11_	Umbilicaxanthoside A
3	C_25_H_28_O_11_	Dracunculifoside K;(-)-Dracunculifoside K
4	C_9_H_14_O_4_	Jioglutolide
**Quinoline**		
1	C_10_H_7_NO_3_	Kynurenic acid
2	C_10_H_7_NO_3_	2-oxo-1,2-dihydroquinoline-4-carboxylic acid

### Effects of *M. subhylanus* meal as bacterial composition modulator in broiler chicks

**Fig. 1:**
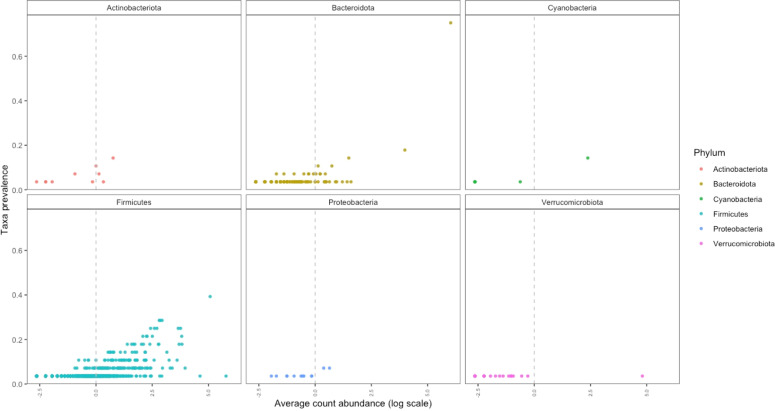
Prevalence of the most dominant bacterial phyla identified in the caecal harvested from Ross 308 broiler chicks fed experimental diets, and the prevalence shown is in relation to the total abundance counts.

**Fig. 2:**
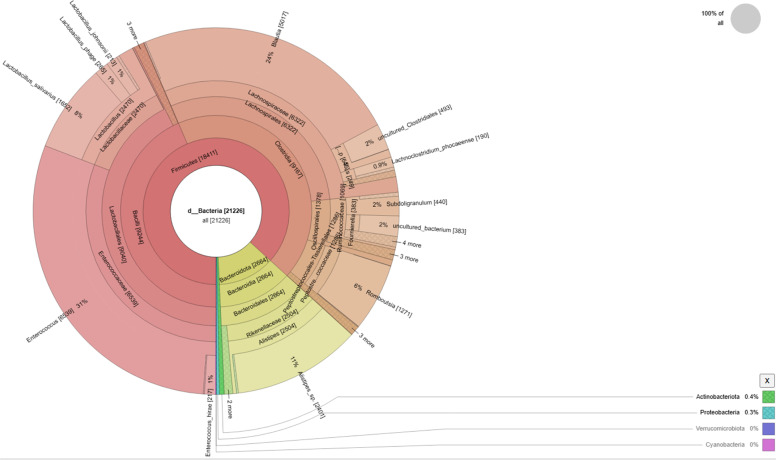

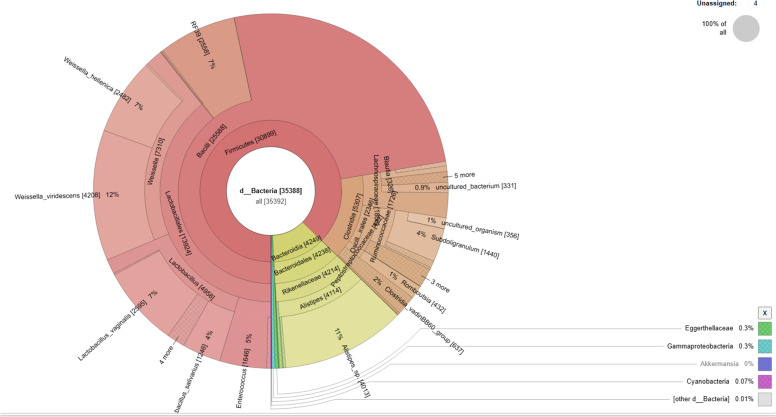

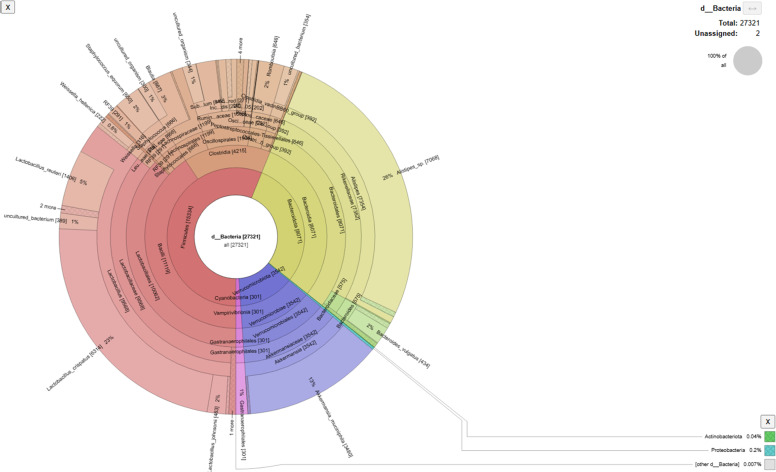
A-C. A Krona plot, depicting the relative abundance of bacterial phylum, genus, and species identified in the broilers fed diets NC, PC, and InsecM.

Total bacterial composition was evaluated from caeca samples on d 14. Six phyla, Actinobacteriota, *Firmicutes, Bacteroidota, Cyanobacteria, Proteobacteria,* and *Verrucomicrobiota,* were most abundant in the caeca contents harvested from broiler chicks fed experimental diets. *Firmicutes* were the most abundant phyla, and *Bacteroidota* were the second most abundant phyla, followed by *Actinobacteriota, Cyanobacteria, Proteobacteria,* and *Verrucomicrobiota*, respectively (Fig. 1). At the genus level, a total of 11 bacteria genus were identified as the most abundant genera in chicks, as shown in (Fig. 2A-C). *Alstipes, Lactobacillus, Akkermansia, Enterococcus, Blautia, Wessella, Romboutsia,* and *RF39* were the most abundant genera on d 14. Most of these genera belonged to the phyla *Firmicutes* and *Bacteroidota*. However, these abundances were accompanied by reductions of *Actinobacteriota, Cyanobacteria, Proteobacteria, Verrucomicrobiota,* and *Vertebrata* in chicks on diets NC and PC.

A Krona plot, depicting the relative abundance of bacterial phylum, genus, and species identified in the broilers fed diets NC, PC and InsecM are shown in Fig. 2A-C, respectively. The chicks on a diet NC had a higher proportion of phyla *Firmicutes* (*Oscillospiraceae, Lactocillus salivarius, Lactobacillus cripatus, and Romboutsia*), followed by *Bacteroidia* (*Alistipes spp, Bacteroides valgatus* and *Bacteroides dorei*), and *Verrucomicrobiota* (*Akkermansia municiphila*), respectively, as indicated in Fig. 2A. However, chicks on a diet PC had a higher proportion of *Firmicutes,* with *Weissella viridescens* accounting for 12%, followed by 7% *Weissella Hellenica,* 7% *Lactobacillus vaginalis,* 5% *Enterococcus*, 4% *Lactobacillus salivarius,* and 4% *Subdoligranulum*, respectively. Furthermore, the chicks on a diet PC had a higher proportion of phyla *Bacteroidia,* with *Alistipes* accounting for 11%, as indicated in Fig. 2B. In contrast, chicks on a diet InsecM had a higher proportion of phylum: *Firmicutes* (*Lactobacillus crispatus, Lactobacillus reuteri, Lactobacillus johnsonii, Blautia, Stophylococcus equorum, Romboutsia, Weissella,* and *Lactobacillus*), *Bacteroidia* (*Alistipes spp* and *Bacteroides vulgatus*) and *Verrucomicrobiota* (*Akkermansia municiphila*), as shown in Fig. 2C.

### Apparent nutrient digestibility and metabolisable energy

The results of the effects of the *M. subhylanus*-based diet in commercial broiler chicks, on apparent DM, CP, EE, and ash digestibility and apparent ME, at the starter phase, are summarised in Table 3. The results revealed that apparent DM digestibility was significantly lower in chicks on treatment NC (83.2%) than in those on a diet containing 0.05 g zinc bacitracin per kg DM and those on a diet containing 100 g of *M. subhylanus* meal per kg DM. Furthermore, CP digestibility was lower (*P* < 0.05) in chicks on a diet NC (83.01%) than those on a diet InsecM, which did not differ (P > 0.05) on a diet PC on day 14. The apparent EE digestibility was not affected (*P* > 0.05) by including either 0.05 g of zinc bacitracin per kg DM and/or 100 g of *M. subhylanus* meal per kg DM in diets. Furthermore, the apparent CF digestibility was significantly lower in chicks on a diet InsecM (75.62%) than those on a diet PC, which did not differ (*P* > 0.05) from those on a diet NC. The ash digestibility and the apparent ME in chicks on a diet InsecM were comparable (*P* > 0.05) to those on diets PC and NC.

**Table 3:** Apparent digestibility on DM, CP, CF, EE, ash, and ME in broiler chicks fed a diet containing 100 g *M. subhylanus* per kg DM at the starter phase (in percentages, unless stated otherwise).

Parameter	1Treatments			2SEM	3*P*-value
	NC	PC	InsecM		
Dry matter	83.2^c^	86.33^a^	85.50^ab^	1.40	0.008
Crude protein	83.01^b^	86.80^a^	86.24^a^	1.62	0.004
Ether extract	90.44	89.27	90.30	2.08	0.51
Crude fibre	83.03^a^	82.08^a^	75.62^b^	2.60	0.03
Ash	55.14	55	58.33	2.69	0.88
Metabolisable energy (MJ/Kg)					
	12.66	12.64	12.67	0.60	0.06

^abc^ Values in the same row, with no common superscript, differ significantly (*P* < 0.05).

1 Treatments: Negative control (NC)= commercial broiler diet, without antibiotics growth promoter (AGP) and insect meal (*M. subhylanus*); (2) Positive control (PC)= commercial broiler diet, with zinc bacitracin (AGP) at 0.05 g; and (3) Insect-based diet (InsecM)= a commercial broiler diet, with 100 g of insect meal per kg DM.

2 Standard error of the mean (SEM).

3 *P*-value= probability value.

### Blood indices parameters

The experimental diets did not affect (*P* > 0.05) the white cell count, segmented heterophil, band heterophil, lymphocyte, monocyte, eosinophil, basophil and morphology of broiler chickens on d 14 (Table 4).

**Table 4:** Effects of dietary zinc bacitracin and insect-based diet on blood profile parameters (in x10^9^/L, unless stated otherwise) in broiler chicks, aged one to 14 days.

Parameter	1Treatments			2SEM	3*P*-value
	NC	PC	InsecM		
White cell count	5.80	5.35	5.60	0.93	0.95
Segmented heterophil	3.51	3.65	4.007	0.67	0.87
Band heterophil	0.13	0.28	0.21	0.13	0.72
Lymphocyte	1.24	0.72	0.74	0.20	0.97
Monocyte	0.38	0.22	0.22	0.13	0.64
Eosinophil	0.18	0.19	0.18	0.08	0.99
Basophil	0.29	0.28	0.23	0.07	0.84
Morphology	2.0	1.67	1.67	0.43	0.82
Packed cell volume (%)	30.67	29.33	30.0	2.01	0.91

1 Treatments: Negative control (NC)= commercial broiler diet, without antibiotics growth promoter (AGP) and insect meal (*M. subhylanus*); (2) Positive control (PC)= commercial broiler diet, with zinc bacitracin (AGP) at 0.05 g; and (3) Insect-based diet (InsecM)= a commercial broiler diet, with 100 g of insect meal per kg DM.

2 Standard error of the mean (SEM).

3 *P*-value= probability value: *P* < 0.05.

### Effects of dietary zinc bacitracin and insect-based diet on blood mineral parameters in broiler chicks on d 14

The experimental diets did not affect (*P* > 0.05) serum minerals (sodium, potassium, chloride, calcium, iron, magnesium) in broiler chicks on d 14 (Table 5).

**Table 5:** Effects of dietary zinc bacitracin and insect-based diet on blood minerals parameters (mmol/l) of broiler chicks (0-14 days).

Parameter	1Treatments			2SEM	3*P*-value
	NC	PC	InsecM		
Sodium	143.33	145.37	145.87	0.80	0.14
Potassium	7.83	5.76	5.64	0.56	0.06
Chloride	111.03	113.80	113.30	1.27	0.33
Calcium	2.38	2.49	2.39	0.08	0.57
Iron	17.53	16.13	16.23	1.60	0.77
Magnesium	0.91	0.89	0.917	0.03	0.83

1 Treatments: Negative control (NC)= commercial broiler diet, without antibiotics growth promoter (AGP) and insect meal (*M. subhylanus*); (2) Positive control (PC)= commercial broiler diet, with zinc bacitracin (AGP) at 0.05 g; and (3) Insect-based diet (InsecM)= a commercial broiler diet, with 100 g of insect meal per kg DM.

2 Standard error of the mean (SEM).

3 *P*-value= probability value.

### Effects of insect-based diet on serum biochemical parameters in broilers

Experimental diets affected (*P* < 0.05) the plasma protein (uric acid) and enzymes (Alanine aminotransferase) profiling only, as shown in Table 6. The results indicate that broiler chicks on a diet PC had lower (*P* < 0.05) uric acid contents (0.17 mmol than those on diets NC and InsecM, which had higher (*P* = 0.04) uric acid contents. Furthermore, the alanine aminotransferase (ALT) level was higher (*P* = 0.005) in chicks on a diet NC (4.40 U/L) than those on diets PC and InsecM, which were similar (*P* > 0.05).

**Table 6:** The effects of dietary AGP and insect-based diet on serum biochemical parameters in broiler chicks at the starter phase.

Parameter	1Treatments			2SEM	3*P*-value
	NC	PC	InsecM		
**Plasma protein profile**					
Total serum protein (TSP) g/l	24.50	23.23	22.07	1.15	0.39
Prealbumin (Pre Alb) g/l	0.17	0.0	0.30	0.13	0.33
Albumin g/l	11.33	11.17	10.53	1.03	0.85
Urea mmol/l	0.41	0.22	0.29	0.07	0.24
Uric acid (UA) mmol/l	0.25^a^	0.17^b^	0.27^a^	0.022	0.04
Total bilirubin (Bili) umol/l	0.0	0.17	0.0	0.07	0.23
**Plasma enzymes profile**					
Aspartate aminotransferase (AST) U/L	503.50	343.93	238.0	93.34	0.21
Alanine aminotransferase (ALT) U/L	4.40^a^	0.37^b^	0.87^b^	0.57	0.005
Amylase U/L	679.17	955.20	864.10	159.74	0.50
Gamma- glutamyl transferase (GGT) U/L	8.433	10.73	10.77	1.09	0.29
**Plasma energy profile**					
Glucose (Gluc) mmol/l	14.03	14.01	13.61	0.53	0.82
Creatine (Create) mmol/l	<18.0	<18.0	<18.0	0.0	-
Cholesterol (Chol) mmol/l	3.59	3.30	3.47	0.34	0.84
Triglycerides (Trig) mmol/l	1.21	0.41	0.88	0.25	0.15

^abc^ Values in the same row, with no common superscript, differ significantly (*P* < 0.05).

1 Treatments: Negative control (NC)= commercial broiler diet, without antibiotics growth promoter (AGP) and insect meal (*M. subhylanus*); (2) Positive control (PC)= commercial broiler diet, with zinc bacitracin (AGP) at 0.05 g; and (3) Insect-based diet (InsecM)= a commercial broiler diet, with 100 g of insect meal per kg DM.

2 Standard error of the mean (SEM).

3 *P*-value= probability value.

### Broiler’s production parameters

The effects of the insect-based diet and zinc bacitracin on feed intake (FI), weight gain (WG) and feed conversion ratio (FCR) were evaluated on d 14 in broiler chicks, as shown in Table 7. Repeated measures analysis revealed significant weeks × diet interaction effects on FI (*P* = 0.001), LW (*P* = 0.003), WG (*P* < 0.001), and FCR (*P* = 0.02). The results revealed that the experimental diets did not affect (*P* > 0.05) FI at 14 days. Furthermore, chicks on a diet InsecM had a higher (*P* < 0.05) WG (483.28 g/bird) than those on a diet NC. However, chicks on diets InsecM and PC had similar (*P* > 0.05) WG. The FCR was lower (*P* *<* *0.05*) in chicks on diets InsecM (1.12) and PC (1.09) than those on a diet NC.

**Table 7:** Growth parameters of broiler chicks fed dietary zinc bacitracin and the insect-based diet.

Parameter	1Treatments			2SEM	3*P*-value
	NC	PC	InsecM		
Feed intake (g/bird)					
	528.50	538.20	541.68	20.50	0.13
Initial weight (g/bird)	53.20	53.85	55.90	1.07	0.22
	501.70^b^	544.78^a^	539.18^a^	7.99	0.03
Weight gain (g/bird)					
	448.50^b^	490.93^a^	483.28^a^	7.67	0.03
Feed conversion ratio					
	1.17^a^	1.09^b^	1.12^b^	0.02	0.01

^abc^ Values in the same row, with no common superscript, differ significantly (*P* < 0.05).

1 Treatments: Negative control (NC)= commercial broiler diet, without antibiotics growth promoter (AGP) and insect meal (*M. subhylanus*); (2) Positive control (PC)= commercial broiler diet, with zinc bacitracin (AGP) at 0.05 g; and (3) Insect-based diet (InsecM)= a commercial broiler diet, with 100 g of insect meal per kg DM.

2 Standard error of the mean (SEM): *P* < 0.05.

3 *P*-value= probability value.

## Discussion

### Bioactive compounds identified in the *M. subhylanus* meal and their properties as natural growth enhancers

Chitin derived from insects has been widely known as a source of secondary metabolites (bioactive compounds), with possible positive effects on the overall health and performance of animals (Malematja et al., 2023). Several reports have demonstrated that chitin contains bioactive compounds with antibacterial, antiviral and antifungal properties, as well as anti-inflammatory and antioxidant activities (Józefiak and Engberg, 2015; Gasco et al., 2018). Recently, there has been increasing interest in edible insect species, such as soldier fly larvae (*Hermetia illucens*), the yellow mealworm (*Tenebrio molitor*), maggot (*Lucilia sericata*), superworm (*Zophobas morio*), house cricket (*Acheta domestica*) and common housefly (*Musca domestica*) in poultry diets, which aims to enhance nutrient digestion, growth performance, and improve the digestive and immune system of birds (Hua et al., 2019; Mabelebele et al., 2023). In the present study, four main classes of bioactive compounds were identified in *M. subhylanus*, which include alkaloids, phenolics, flavonoids and quinoline. These classes of bioactive compounds are believed to possess multiple functions, such as an immune modulatory effect, containing anti-microbial agents, and antioxidant and anti-inflammatory properties (Xu et al., 2017). The alkaloid compounds include Hyacinthacine C1; (+)-Hyacinthacine C1, N—Hydroxyannomontine and Floripavidine. These compounds are known to have anti-inflammatory properties (Li et al., 2020). The identified phenolics include the compounds phellodendric acid A; (+)-Phellodendric acid A and Umbilicaxanthoside A, which possess antioxidant and anti-inflammatory properties, as well as health-promoting properties, such as anti-bacterial, anti-carcinogenic and anti-pathogenic microorganisms (Dehhaghi et al., 2020). Rosmarinic acid, one of the flavonoid compounds identified in the present study, is known to have antioxidant and anti-inflammatory properties (Xu et al., 2017). Kynurenic acid, which belongs to the quinoline metabolites, regulates the metabolism that modulates gut microbiota, including the bacterial phyla, *Actinobacteria, Bacteroides, Firmcutes* and *Fusobacteria,* in hosts in animals (Dehhaghi et al., 2020). With these properties, the obtained results suggest that the *M. subhylanus* meal could be a potential novel antimicrobial agent, immune modulator and growth enhancer in poultry and/or other farm animals. Phenolics, quinoline and flavonoids play a significant role in immunomodulation and resistance to various diseases, as well as in maintaining the proportional balance between the gut microflora (Rivas-Navia et al., 2023; Torres-Castillo et al., 2023). These claims are supported by the study by Sogari et al. (2019), Ndotono et al. (2022) and Vasilopoulos et al. (2024), who reported that phyla increased the proportion of beneficial gut microflora and modulated the immune system in birds fed diets containing black soldier fly and yellow mealworm. These findings could mean that adding *M. subhylanus* to broiler diets could possibly stimulate gut development and the immune system, and, subsequently, improve the overall health condition and performance of the bird. By taking the biological value and functions of alkaloids, flavonoids, phenolics and quinoline, and their possible positive health effects into consideration, dietary *M. subhylanus* was formulated for broiler chicks, which was expected to lay the foundation for follow-up research, to test the efficacy of substituting AGP with insect meal in the diets for broilers.

### Bacterial composition in the intestinal gut of broiler chicks on d 14

A considerable number of studies have documented that insects contain various biological chemical compounds, such as alkaloids, flavonoids and phenolic acids, with verified antibacterial, antioxidant and anti-inflammatory properties, which could have a similar effect on gut microbiome as commercial antibiotics (Roos and Van Huis, 2017; Nino et al., 2021). The Gut microbiota has been proven to be beneficial in growth activities and the overall health condition of hosts (Yang et al., 2023). It is, therefore, important to compare the effect of insect meal and commercial antibiotics growth promoters on intestinal microbiota modulation. In the present study, the results revealed that bacterial diversity was higher in broiler chicks on an InsecM diet, followed by those on a PC diet. *Firmicutes* were the predominant phylum, proportionate to *Actinobacteriota, Bacteroidota, Cyanobacteria, Proteobacteria, Verrucomicrobiota,* and *Vertebrata* in broiler chicks on an InsecM diet during the starter phase. Furthermore, *Firmicutes* diversity was predominantly higher in broiler chicks on InsecM, followed by those on the PC diet. The dominant *Firmicutes* families in broiler chicks on treatment InsecM, were *Lactobacillus crispatus, Lactobacillus reuteri, Lactobacillus vaginalise, Lactobacillus salvarius, Blautia spp, Stophylococcus equorum, Romboutsia, Weissella hellenica,* and *Weissella viridescens*. Bacteria belonging to these genera have been documented to be beneficial bacteria, involved in many metabolic processes in hosts (Ndotono et al., 2022). Most of the *Firmicutes* families are used as prebiotics; for instance, *Lactobacillus reutri* and *Lactobacillus crispatus* are known to modulate intestinal innate mucosal immunity (Sogari et al., 2019), and *Romboutsia* regulates activities of the intestinal digestive enzymes, as well as fermenting carbohydrates and single amino acids (Kumar et al., 2018).

In addition, the abundance of *Lactobucillus crisptus* and *johnsonii* bacteria detected in broiler chicks on InsecM diet plays a crucial role in digestion and nutrient absorption, as well as maintaining the relationship between the gut microbiota and the health of the host (Rychlik, 2020; Zhang et al., 2023). Genus *Alistipes spp,* from the family *Rikenellaceae* and belonging to the phylum *Bacteroidia,* are known to be among the first colonizers (Rychlik, 2020) and were also higher across the experimental diets. *Alistipes spp* are beneficial gut microbes that are bile resistant, which regulate pathogenic metabolites, digest gelatine and ferment carbohydrates (Kumar et al., 2018); thus, improving gut development and nutrient utilisation. Furthermore, phylum *Verrucomicrobiota* became dominant in broiler chicks that were on diets NC and InsecM. *Akkermansia municiphila,* from phylum *Verrucomicrobiota,* were predominant in chicks on diets NC and InsecM; this genus exerts a beneficial effect on gut integrity improvement and inflammatory alleviation and regulates metabolite processes in hosts (Yang et al., 2022). Therefore, the results suggest that utilising *M. subhylanus* meal in broiler diets favours the abundance of beneficial intestinal microbiota, which, in turn, could improve the gut development and immune system of the host. This implies that a diet having 100 g *M. subhylanus* meal per kg DM is a suitable substitute for zinc bacitracin, since no significant changes were observed in microbial communities. In addition, the results are in line with studies by Sogari et al. (2019), Ndotono et al. (2022), and Vasilopoulos et al. (2024), who reported phyla *Firmicutes* and *Bacteroidota* as the most predominant in broilers fed diets containing incremental levels of black soldier fly and yellow mealworm. The similarities observed in microbial communities in broiler chickens fed dietary *M. subhylanus* meal and zinc bacitracin may lead to a shift away from the use of commercial antibiotic growth promoters in broilers’ diets. In contrast, Kumar et al. (2018) reported a low abundance of *Firmicutes* and predominant *Bacteroidetes* in birds. The possible explanation for the variation in the results could be associated with the type of diet, bird species and the age of the bird at data collection.

### Apparent nutrient digestibility and apparent metabolisable energy

The apparent nutrient digestibility and metabolisable energy reflect the degree of absorption and utilisation of the feedstuff (Chu et al., 2020). Previously, insect meals were evaluated as an excellent source of bioactive compounds, which are believed to aid in nutrient digestion (Malematja et al., 2023). The presence of alkaloids, flavonoids, phenolics and quinoline compounds suggested the effective utilisation of insect meals in poultry diet formulation for better nutrient digestion and growth. In the present study, a decrease in the apparent CF digestibility was observed in broiler chicks that were on an InsecM diet on d 14. Possible explanations for the lower CF digestibility observed in broiler chicks on InsecM could be due to the presence of chitin in *M. subhylanus* meal and the fact that, at an early age, broiler chicks have an underdeveloped digestive system, so they might have overly sensitive to the presence of chitin in the diet and unable to utilise it efficiently (Gajana et al., 2011). Improved CP digestibility in broiler chicks that were on an InsecM diet could be explained by the observed abundance of *Alistipes spp, Lactobacillus crispatus, Lactobacillus reuteri, Akkermansia municiphila,* and *Blautia spp*. These bacteria are beneficial gut microbes and are known to aid in nutrient fermentation and digestion (Kumar et al., 2018); hence, improved CP digestibility. The improvement in CP apparent digestibility suggests that broiler chicks were able to utilise *M. subhylanus* more efficiently. In addition, observed results indicate that the InsecM diet reflected comparative EE, ash, and ME apparent digestibility values to dietary zinc bacitracin. Therefore, this implies that a diet containing 100 g *M. subhylanus* meal per kg DM could be adopted as an alternative feed additive to enhance nutrient digestibility in poultry. These results are in line with those reported by Attia et al. (2023), who observed increased apparent CP digestibility in birds that are fed a diet containing insect meals.

### Blood indices and serum parameters

Haematology and serum parameters are good indicators of the physiological state of the animal, and their variation is used to diagnose clinical diseases (Onasanya et al., 2015). Additionally, blood analysis parameters are used to monitor the impact of therapeutic and nutritional interventions in animals (Peres et al., 2015). Blood analysis is also used to determine the health status of the animal, detect chronic diseases that do not show clinical symptoms and ascertain nutrient utilisation by the animal (Manyeula et al., 2019). Therefore, haematological and serum biochemical analyses are routine practices for livestock to provide the health and physiological status of the animal. In the current study, an insect-based diet did not affect haematological parameters at the starter phases. This is in line with the results discovered by Dabbou et al. (2018) and Bellezza et al. (2021), who observed no effect on haematological parameters in broilers fed diets containing black soldier fly meal and yellow mealworm, thus confirming our findings. The lack of significant difference in haematological analyses at the starter phases suggests that a diet having 100 g of *M. subhylanus* meal per kg DM could be safely used in broiler diets, without affecting the metabolic and health status of the birds.

Notably, Mat et al. (2022) reported alleviated lymphocytes, packed cell volume and elevated monocytes in broilers that are fed an incremental level of black soldier fly meal. Furthermore, the dietary *M. subhylanus* diet did not affect serum biochemical parameters, except for plasma protein (uric acid) and enzymes (Alanine aminotransferase) profiling. In the current study, an InsecM diet resulted in elevated uric acid levels compared to PC on day 14. Uric acid is a major end product of exogenous and endogenous purine metabolism (Ding et al., 2021). Elevated concentrations of uric acid indicate poor protein utilisation by the birds (Ogunwole et al., 2017), suggesting that including 100 g of insect meal per kg DM contains enough anti-nutritional factors which affected catabolism of the protein. Chicks on an InsecM diet had relatively lower levels of ALT concentration; however, the values obtained in the present study were within the normal range, namely 0-10 IU/L, which is acceptable for chickens (dos Santos Schmidt et al., 2009). Alanine aminotransferase is a sensitive tool indicator of liver disease in chickens (Kokore et al., 2021). Therefore, the obtained ALT values in the present study indicate the absence of cell injuries in the liver, and the findings in this study confirm that an InsecM diet did not damage the liver and other vital organs. This is in line with the findings of a study by Mat et al. (2022), who reported unaffected ALT in broilers fed dietary insect meal.

### Broiler’s production parameters

Regardless of the nature of fibre content present in the *M. subhylanus* meal, the present results suggest that broiler chicks were able to utilise insect meal efficiently, without adverse effects on their FI during the starter phase. In addition, the effects of *M. subhylanus* on growth performance parameters could be linked to the modification of intestinal microbes. The effects of the abundance of beneficial bacteria and improved CP digestibility observed in the broiler chicks that were on an InsecM diet were reflected in the growth performance parameters, including WG and FCR, which compared well with those that were on dietary zinc bacitracin (positive control; PC). Another possible explanation for improved growth parameters in chicks that were on treatment InsecM, compared to those that were on NC, could be due to health benefits secondary metabolites identified in *M. subhylanus -* which is in line with the work done by Musundire et al. (2016) and Sailo et al. (2020) and highly digestible amino acids (Bovera et al., 2015; Mabelebele et al., 2023). Therefore, these observations could mean that adding up to 100 g of *M. subhylanus* to a commercial broiler diet has the potential to substitute the commercial growth promoter (zinc bacitracin). The results of the present study concur with studies conducted by Islam and Yang (2016), Benzertiha et al. (2020), and Sedgh‐Gooya et al. (2022), who reported increased LW and BWG, as well as improved FCR in broiler chickens that are fed diets containing either *Tenebrio molitor* or *Zophobas morio*. However, the results of the current study are inconsistent with the results obtained by Olonde et al. (2023), in which AFI in indigenous chickens was reduced by 5% inclusion of *Macrotermes bellicosus*. It was suggested that insect meal, at higher inclusion levels, reduced AFI because of the lipid and protein content (Moyo et al., 2020). The inconsistent findings could be associated with insect species, poultry breeds and the age of the birds.

### Limitation of the study

Edible insect meals contain chitin and other antinutritional factors, which might interfere with nutrient digestibility in broiler chickens. Furthermore, insects are known to possess heavy metals, which might become toxic when fed to chickens (Mabelebele et al., 2023; Malematja et al., 2023). Concerns about the viability of *M. subhylanus* and/ or other edible insect species as a potential feed additive are on the rise (Khalifah et al., 2023).

## Conclusion and recommendations

The efficacy of substituting commercial antimicrobial/ antibiotic growth promoters with insect meal in broiler diets, as a gut microbiome and immune system modulator, feed additive, and growth enhancer, has been studied. Metabolite analysis results indicate that *M. subhylanus* possesses valuable secondary metabolites, which are known to act as antimicrobial, gut bacterial composition modulators, and growth enhancers. Furthermore, the present study evaluated the effects of using 100 g of alate termite (*M. subhylanus*) meal per kg DM as a potential growth enhancer/ modulator on cecal bacteria composition, blood haematology and serum, nutrient digestibility, and growth performance. Obtained results demonstrated that broiler chickens on a diet having 100 g of *M. subhylanus* per kg DM increased an abundance of beneficial bacterial composition, belonging to phyla *Firmicutes, Bacteroidota,* and *Verrucomicrobiota* and also improved CP digestibility in broiler chicks on d 14. In addition, the inclusion of 100 g *M. subhylanus* meal per kg DM affected uric acid and ALT. The obtained results also indicated that broiler chickens on a diet having 100 g of *M. subhylanus* meal/kg DM, and those on a diet supplemented with 0.05 g of zinc bacitracin per kg DM had similar effects on WG and FCR. However, since there is little literature available on the effect of including *M. subhylanus* meal in broiler diets, further studies are encouraged to confirm these findings. Moreover, studies investigating the effects of the *M. subhylanus* on gut microbial community modulation, gut morphology, haematology, and growth performance in broiler chickens, at the grower and finisher phase, aim to understand the mode of action by which *M. subhylanus* stimulates growth in broiler chickens, which would be of great importance in addition to future studies.

## Data availability

The dataset(s) supporting the conclusions of this article are available in the NCBI Sequence Read Archive (SRA) repository, Accession number: PRJNA1237245.

## Funding

This study was funded by the National Research Foundation (grant number PMDS22052715122) and Insect Project (ASDG-RSP).

## CRediT authorship contribution statement

**Emmanuel Malematja:** Writing – review & editing, Writing – original draft, Visualization, Methodology, Formal analysis, Data curation, Conceptualization. **Thendo Mafuna:** Formal analysis, Data curation. **Nthabiseng Amenda Sebola:** Writing – review & editing, Supervision. **Sekobane Daniel Kolobe:** Validation, Data curation, Conceptualization. **Monnye Mabelebele:** Validation, Supervision, Project administration, Methodology, Data curation, Conceptualization.

## Disclosures

The authors reported no potential conflict of interest.

## Appendix Supplementary materials

Image, application 1

## Footnotes

Supplementary material associated with this article can be found, in the online version, at doi:10.1016/j.psj.2026.107218.
