# Supplementary material for: Effects of replacing commercial zinc bacitracin with insect meal (Macrotermes subhylanus) on caeca bacteria composition, haematology, and growth performance in commercial broiler chicks at the starter phase
Source: Poult Sci. 2026 Jun 2;105(9):107218. doi: 10.1016/j.psj.2026.107218 (PMC13292562; doi:10.1016/j.psj.2026.107218)
Supplement: Supplementary file 1 [file mmc1.docx]

**Table S1**. Proximate analysis of winged termite meal (*M. subhylanus*) in percentage on a dry matter basis

| **Nutrient** | **Composition (%)** |
| --- | --- |
| Dry matter | 95.80 |
| Crude protein | 53.62 |
| N-average | 8.58 |
| Crude fibre | 6.83 |
| EE | 22.43 |
| Ash | 9.68 |
| NDF | 27.23 |

N-average=nitrogen average, EE=ether extracts, NDF=neutral detergent fibre

**Table S2. Mineral and proximate analysis of the experimental diets on dry matter (in %, unless stated otherwise)**

| **Nutrient** | **Composition** | | |
| --- | --- | --- | --- |
|  | **NC** | **PC** | **IBD** |
| Ash | 4.24 | 4.24 | 4.91 |
| Crude fibre | 4.25 | 4.25 | 4.45 |
| Moisture |  |  |  |
| Crude protein | 22 | 22 | 22 |
| Ether extract | 7.08 | 7.12 | 4.99 |
| Metabolisable energy (MJ/ kg DM) | 12 | 12 | 12 |
| Calcium | 0.9 | 0.9 | 0.96 |
| Phosphorous | - | - | - |

Negative control (**NC**)= commercial broiler diet, without antibiotics growth promoter (AGP) and insect meal (*M. subhylanus*); **(2)** Positive control **(PC**)= commercial broiler diet, with zinc bacitracin (AGP) at 0.05%; and **(3)** Insect-based diet (**IBD**)= a commercial broiler diet, with 10 g/kg insect meal (*M. subhylanus*).
